# Overexpression of serum extracellular vesicle microRNA-215-5p is associated with early tumor recurrence and poor prognosis of gastric cancer

**DOI:** 10.6061/clinics/2021/e2081

**Published:** 2021-04-26

**Authors:** Yunfei Zhang, Fengchang Huang, Ning Xu, Jin Wang, Dan Li, Liang Yin

**Affiliations:** I Department of Oncology, The First Affiliated Hospital of Kunming Medical University, Kunming 650032, Yunnan, China.; II Department of Vascular Surgery, The First Affiliated Hospital of Kunming Medical University, Kunming 650032, Yunnan, China.; III Department of Acupuncture, Yunnan Hospital of Integrated Traditional Chinese and Western Medicine, Kunming 650200, Yunnan, China.

**Keywords:** Gastric Cancer, Serum Extracellular Vesicle miR-215-5p, Early Tumor Recurrence, Prognosis, Diagnosis

## Abstract

**OBJECTIVES:**

Extracellular vesicle microRNAs (EV-miRNAs) have been demonstrated to be reliable candidate biomarkers for clinical applications. However, the clinical application potential of serum EV-miR-215-5p for gastric cancer (GC) remains poorly understood. The goal of our study was to determine the efficacy of serum EV-miR-215-5p in predicting the prognosis of GC.

**METHODS:**

Blood samples were collected from 118 patients with GC, 60 patients with benign gastric disease and BGD and 70 healthy controls. The relative levels of serum EV-miR-215-5p were measured using quantitative real-time polymerase chain reaction (qRT-PCR).

**RESULTS:**

Compared to patients with BGD and normal controls, GC patients exhibited remarkably higher serum EV-miR-215-5p level, especially those with early tumor recurrence (ETR). Receiver operating characteristic (ROC) curve analysis showed that serum EV-miR-215-5p was able to distinguish GC patients from BGD patients or healthy controls and GC patients with ETR from those without ETR. In addition, increased serum EV-miR-215-5p levels were notably correlated with invasive depth, TNM stage, and lymph node metastasis. Moreover, serum EV-miR-215-5p levels were greatly decreased after surgical treatment, but increased at the time of ETR. Survival analysis showed that patients with higher serum EV-miR-215-5p had shorter survival. Furthermore, serum EV-miR-215-5p was an independent risk factor for GC.

**CONCLUSIONS:**

Serum EV-miR-215-5p might be a novel biomarker for predicting ETR and prognosis of GC.

## INTRODUCTION

Gastric cancer (GC) is the fourth most common malignancy worldwide, and owing to its high mortality rates, it has become a global human health problem (1,2). Early diagnosis and appropriate treatment of GC are undoubtedly the best strategies for improving survival. Unfortunately, detection of GC at the initial stage is very difficult because of its asymptomatic nature. Thus, many GC patients are diagnosed at advanced stages of the disease characterized by invasion and metastasis (3,4). Accurate and efficient monitoring of the development of GC considerably contributes to ameliorating its poor prognosis (5). Early tumor recurrence (ETR) is an important predictor of unfavorable prognosis in GC. Therefore, there is a compelling need to identify robust biomarkers for predicting the ETR and prognosis of GC.

MicroRNAs (miRNAs) are a class of well-conserved non-coding RNAs (approximately 19-24 nucleotides in length) (6) that are involved in cancer development, including proliferation, invasion, metastasis, and apoptosis of cancer cells. miRNAs may function either as tumor promoters or suppressors, depending on the downstream targets they regulate (7,8). Extracellular vesicles (EVs) include exosomes and microvesicles and can be found in the blood, urine, or other bodily fluids (9). Recent studies have demonstrated that extracellular vesicle miRNAs (EV-miRNAs) can be stably detected in peripheral blood and protected against degradation by enzymes (10). Therefore, serum EV-miRNAs can potentially be employed for GC diagnosis and prognosis. For instance, serum exosomal miR-423-5p is overexpressed in GC, and its upregulation is strongly associated with lymph node metastasis (11). Similarly, serum exosomal miR-1246 levels were also markedly increased in GC (12).

miR-215-5p has been previously identified as an oncomiR in GC (13-15). However, the potential clinical value of serum EV-miR-215-5p in GC is poorly understood. In this prospective study, we aimed to assess the expression pattern and prognostic value of serum EV-miR-215-5p in patients with GC.

## MATERIALS AND METHODS

### Patients and samples

This study was approved by the Ethics Committee of The First Affiliated Hospital of Kunming Medical University. Written informed consent was obtained from all the participants before serum sample collection. A total of 118 patients diagnosed with GC, 60 with benign gastric disease (BGD), and 70 healthy donors were recruited. All patients with GC underwent gastrectomy. The clinical stage was evaluated in strict accordance with the Union for International Cancer Control (UICC) tumor-node-metastasis (TNM) 7^th^ edition classification. None of the patients received any therapy before blood sample collection. ETR was defined as recurrence within a year after the surgery, and a total of 29 patients with GC showed ETR. Detailed clinical information of all GC patients is presented in Table 1.

Peripheral blood was collected from all participants. The samples were then centrifuged in serum-separator tubes at 3,000 x *g* for 10 min. The supernatant was transferred to a fresh microfuge tube and stored at -80°C until further use. Blood samples were obtained from all patients with GC two weeks after the surgical treatment and from patients with ETR at the time of ETR.

### Extracellular vesicle isolation

EVs were extracted from serum samples using the ExoQuick Exosome Precipitation Solution (System Biosciences, Mountain View, CA, USA) according to the manufacturer’s instructions. Briefly, followed by centrifugation at 3,000 x *g* for 15 min, the supernatant of the thawed serum was mixed with 1/4 volume of ExoQuick solution. The mixture was incubated for 30 min at 4°C and then centrifuged at 1,500 x *g* for 30 min. EV-enriched pellets were dissolved in PBS and stored at -80°C for further analysis.

### Quantitative real-time polymerase chain reaction (qRT-PCR)

The mirVana PARIS Kit (Ambion, Austin, TX, USA) was used to extract total RNA from the EVs. RNA quality was checked on a Nanodrop ND-1000 spectrophotometer (Nanodrop Technologies, Wilmington, DE, USA). Total RNA was reverse transcribed into complementary DNA (cDNA) using the TaqMan MicroRNA Reverse Transcription Kit (Applied Biosystems, Foster City, CA, USA). EV- miR-215-5p expression was analyzed using SYBR¯ Premix Ex Taq™ II (Takara, Dalian, China) and cDNA, and the reaction was run on a 7000 Real-Time PCR system (Applied Biosystems). Each reaction was performed in triplicate. Cel-miR-39 was used as the external control, and the relative serum EV-miR-215-5p level was calculated using the 2^-ΔΔCt^ method.

### Western blot

The proteins were resolved on a 10% SDS-PAGE gel and transferred onto a polyvinylidene difluoride membrane. The membranes were blocked with 5% skim milk for 1h at room temperature (25°C), and then probed with primary antibodies against TSG-101 (1:500 dilution, Abcam, Cambridge, UK) and CD9 (1:500 dilution; Abcam) in a cold room overnight. Subsequently, the membranes were incubated with horseradish peroxidase-linked secondary antibodies for 1h at room temperature. Bands were visualized using enhanced chemiluminescence reagents (GE Healthcare, Piscataway, NJ, USA).

### Statistical analysis

The Mann-Whitney U test or Kruskal-Wallis test were performed to evaluate the differences between or among the groups with respect to serum EV-miR-215-5p levels. The correlations between serum EV-miR-215-5p levels and clinicopathological variables were assessed using the Chi-square test. Receiver operating characteristic (ROC) curve and area under the curve (AUC) were used to determine the diagnostic performance of serum EV-miR-215-5p. Overall survival (OS) and disease-free survival (DFS) were assessed using Kaplan-Meier curves and log-rank tests. Univariate and multivariate analyses were performed to identify the independent prognostic factors for GC. Significance was set at *p* ≤0.05. GraphPad Prism (version 5.01; GraphPad Software, San Diego, CA, USA) and MedCalc version 12.3.0 (MedCalc, Mariakerke, Belgium) were used for statistical analyses.

## RESULTS

### Upregulation of serum EV-miR-215-5p in GC

**Figure 1:**
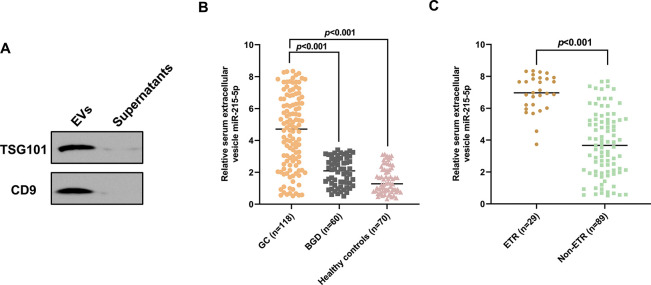
Serum EV-miR-215-5p level was higher in GC patients. (A) The isolated extracellular vesicles were positive for TSG-101 and CD9. (B) Serum extracellular vesicle miR-215-5p levels were markedly higher in GC patients than those in BGD patients and healthy subjects. (C) Serum extracellular vesicle miR-215-5p levels were higher in patients with ETR than in those without ETR.

Western blotting revealed that the serum-derived EVs were positive for EV markers TSG-101 and CD9, while these were expressed at very low levels in the supernatants (Figure 1A). qRT-PCR was then used to measure the expression of serum EV-miR-215-5p in all participants. As presented in Figure 1B, serum EV-miR-215-5p levels were markedly elevated in GC patients than those in BGD patients and healthy controls (***p<0.001). Moreover, significantly higher serum extracellular vesicle miR-215-5p levels were observed in GC patients with ETR than in those without ETR (***p<0.001, Figure 1C).

### Diagnostic performance of serum EV-miR-215-5p in GC

**Figure 2:**
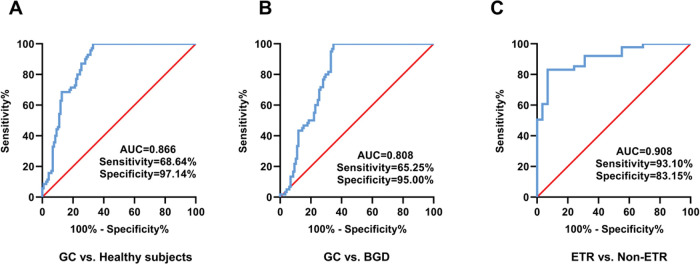
The diagnostic performance of serum EV-miR-215-5p. (A) The diagnostic performance of serum EV-miR-215-5p for distinguishing GC patients from healthy donors. (B) The diagnostic performance of serum EV-miR-215-5p for distinguishing GC patients from BGD patients. (C) The diagnostic performance of serum EV-miR-215-5p for identifying GC patients with ETR from those without ETR.

As shown in Figure 2A, serum EV-miR-215-5p distinguished GC patients from healthy donors, with an AUC value of 0.866 (sensitivity=68.64%, specificity=97.14%), and the serum EV-miR-215-5p distinguished GC patients from BGD patients with an AUC of 0.808 (sensitivity=65.25%, specificity=95.00%) (Figure 2B). Importantly, serum EV-miR-215-5p exhibited an AUC of 0.908 (sensitivity=93.10%, specificity=83.15%) for distinguishing GC patients with ETR from GC patients without ETR (Figure 2C).

### Correlation of serum EV-miR-215-5p with clinical variables in GC

All 118 GC patients were stratified into the high serum EV-miR-215-5p group (n=59) and low serum EV-miR-215-5p group (n=59) based on the median expression of serum EV-miR-215-5p. High serum EV-miR-215-5p levels were positively correlated with invasive depth (*p*=0.0032), TNM stage (*p*<0.0001), and lymph node metastasis (*p*=0.0008). However, serum EV-miR-215-5p was not correlated with sex (*p*=0.1338), age (*p*=0.1648), histological type (*p*=0.1951), or distant metastasis (*p*=0.0586).

### Dynamic changes in serum EV-miR-215-5p levels after gastrectomy

**Figure 3:**
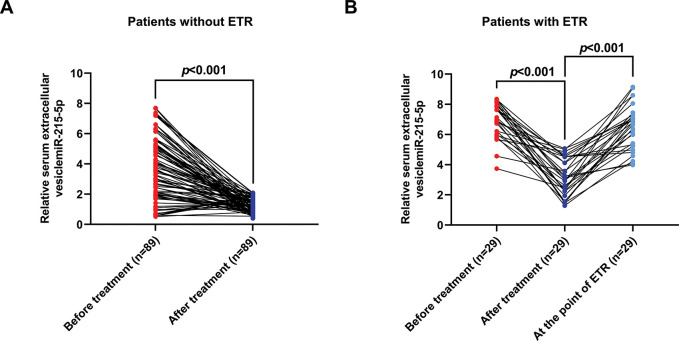
The serum EV-miR-215-5p level was sensitive to treatments. (A) Serum EV-miR-215-5p level was significantly decreased in GC patients without ETR after gastrectomy. (B) The serum EV-miR-215-5p level was decreased in GC patients with ETR after gastrectomy but increased at the point of ETR.

Compared to pre-treatment blood samples, blood samples from patients with ETR and those without ETR exhibited significantly decreased levels of serum EV-miR-215-5p (***p<0.001) (Figure 3A-3B). In patients with ETR, serum EV-miR-215-5p levels were dramatically elevated when ETR occurred (***p<0.001) (Figure 3B).

### Correlation of serum EV-miR-215-5p level with OS and DFS

**Figure 4:**
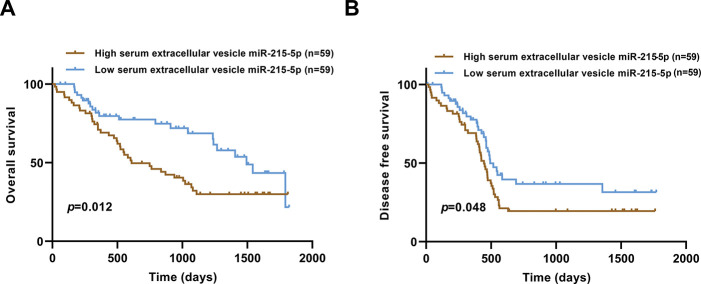
Correlation between serum EV-miR-215-5p level and OS and DFS. (A) GC patients with high serum EV-miR-215-5p levels had shorter OS. (B) GC patients with higher serum EV-miR-215-5p levels had worse DFS.

The prognostic differences in terms of OS and DFS were evaluated using Kaplan-Meier survival curves. As shown in Figure 4A, GC patients in the high serum EV-miR-215-5p group had shorter OS (*p*=0.012). Likewise, patients with higher serum EV-miR-215-5p levels tended to have a low DFS (*p*=0.048, Figure 4B).

### Cox regression analysis of the factors affecting OS

Univariate analysis demonstrated that invasive depth (HR=2.65, 95% CI=1.12-4.35, *p*=0.036), lymph node metastasis (HR=3.12, 95% CI=1.48-6.09, *p*=0.008), TNM stage (HR=3.98, 95% CI=1.87-8.97, *p*<0.001), and serum EV-miR-215-5p levels (HR=2.95, 95%CI=1.31-5.68, *p*=0.013) were significantly associated with OS in GC. Multivariate analysis revealed that lymph node metastasis (HR=2.71, 95% CI=1.36-5.32, *p*=0.012), TNM stage (HR=3.67, 95%CI=1.61-7.58, *p*=0.003), and serum EV-miR-215-5p levels (HR=2.28, 95% CI=1.23-4.36, *p*=0.028) were independent prognostic factors for GC (Table 2).

## DISCUSSION

To our knowledge, this was the first study to evaluate the clinical significance of serum EV-miR-215-5p levels in GC. First, we found that serum EV-miR-215-5p levels were elevated in GC patients, especially in patients with ETR. Second, ROC analysis revealed that serum EV-miR-215-5p was a promising biomarker for distinguishing GC patients with ETR from those without ETR. Third, a strong association was found between high serum EV-miR-215-5p levels and unfavorable clinical outcomes. Fourth, the serum EV-miR-215-5p level was sensitive to therapeutic responses. Finally, the serum EV-miR-215-5p was an independent prognostic factor for GC. In conclusion, serum extracellular vesicle miR-215-5p may serve as a non-invasive biomarker for predicting the prognosis of GC and monitoring therapeutic responses.

Consistent with our results, miR-215-5p expression is significantly upregulated in GC tissues. Overexpression of miR-215-5p greatly stimulates the migration and invasion of cancer cells by resulting in the degradation of FOXO1 (13). Similarly, upregulation of miR-215-5p is observed in GC tissues and cell lines, which results in significantly enhanced carcinogenesis (14). miR-215-5p expression is significantly increased in GC tissues and cell lines than that in their respective controls. Ectopic expression of miR-215-5p dramatically promotes the malignancy of GC cells, and *vice versa* (15,16). miR-215-5p upregulation occurs more frequently in high-grade glioma, and miR-215-5p overexpression has been positively correlated with poor prognosis. In addition, *in vitro* analysis showed that enforced miR-215-5p expression promotes migration and invasion of glioma cells by targeting RB1, and *vice versa* (17,18).

In contrast, miR-215-5p may also play a tumor-suppressive role in various cancer types. miR-215-5p expression is significantly lower in non-small cell lung cancer (NSCLC) cell lines and tissues. Upregulation of miR-215-5p suppressed the proliferation, migration, and apoptosis of NSCLC cells *in vitro*, and inhibited tumorigenesis *in vivo* (19-21). miR-215-5p levels are decreased in acute myeloid leukemia, and its downregulation is associated with shorter survival (22). Similarly, miR-215-5p is underexpressed in papillary thyroid cancer (PTC) tissues and cell lines. Enforced miR-215-5p expression dramatically limits the tumorigenicity of cancer cells by regulating the expression of ARFGEF1 (23). MiR-215-5p is frequently downregulated in epithelial ovarian cancer (EOC) tissues and cell lines. Overexpression of miR-215-5p significantly suppresses EOC progression both *in vitro* and *in vivo* (24,25).

In summary, serum EV-miR-215-5p levels are elevated in GC, and their upregulation is strongly associated with poor prognosis in GC. Therefore, serum EV-miR-215-5p may serve as a novel and robust biomarker for stratifying GC patients with different outcome risks.

## AUTHOR CONTRIBUTIONS

Zhang Y and Yin L designed the study and supervised the experiments. Zhang Y, Huang F, Xu N, Wang J, Li D, and Yin L collected the data, performed the experiments, analyzed the data, and wrote the manuscript.

## Figures and Tables

**Table 1:** Association between serum EV-miR-215-5p levels and clinical parameters of GC.

Parameters	N=118	Serum extracellular vesicle miR-215-5p		*p*
		Low	High	
Sex				0.1338
Male	70	39	31	
Female	48	20	28	
Age (years)				0.1648
<60	81	44	37	
≥60	37	15	22	
Histological type				0.1951
Intestinal	53	30	23	
Diffuse	65	29	36	
Distant metastasis				0.0586
No	96	52	44	
Yes	22	7	15	
Invasive depth				0.0032
T1/T2	58	37	21	
T3/T4	60	22	38	
Lymph node metastasis				0.0008
No	52	35	17	
Yes	66	24	42	
TNM stage				<0.0001
I/II	55	41	14	
III/IV	63	18	45	

**Table 2:** Univariate and multivariate analysis to identify an association between clinical parameters and OS of GC patients.

Parameters	Univariate analysis			Multivariate analysis		
	HR	95% CI	*p*	HR	95% CI	*p*
Sex	1.06	0.71-1.94	0.506	-	-	-
Age	1.14	0.46-2.12	0.722	-	-	-
Histological type	0.69	0.43-1.37	0.348	-	-	-
Distant metastasis	1.42	0.87-2.20	0.271	-	-	-
Invasive depth	2.65	1.12-4.35	0.036	-	-	-
Lymph node metastasis	3.12	1.48-6.09	0.008	2.71	1.36-5.32	0.012
TNM stage	3.98	1.87-8.97	<0.001	3.67	1.61-7.58	0.003
Serum EV-miR-215-5p	2.95	1.31-5.68	0.013	2.28	1.23-4.36	0.028
